# Surgical Timing and Safety of Breast Cancer Operations After COVID-19: A Prospective-Only Meta-Analysis of Cohort Studies

**DOI:** 10.3390/jcm15010341

**Published:** 2026-01-02

**Authors:** Ioana-Georgiana Cotet, Diana-Maria Mateescu, Dragos-Mihai Gavrilescu, Andrei Marginean, Stefania Serban, Adrian-Cosmin Ilie, Cristina Guse, Ana-Maria Pah, Marius Badalica-Petrescu, Stela Iurciuc, Maria-Laura Craciun, Adina Avram, Cristina Tudoran

**Affiliations:** 1 Department of General Medicine, Doctoral School, “Victor Babes” University of Medicine and Pharmacy, Eftimie Murgu Square 2, 300041 Timisoara, Romania; ioana.cotet@umft.ro (I.-G.C.); diana.mateescu@umft.ro (D.-M.M.); stefania.serban@umft.ro (S.S.); cristina.marin@umft.ro (C.G.); 2 Centre of Molecular Research in Nephrology and Vascular Disease, University of Medicine and Pharmacy “Victor Babes” Timisoara, Eftimie Murgu Square, Nr. 2, 300041 Timisoara, Romania; tudoran.cristina@umft.ro; 3 Department of Orthodontics, Dental District, Strada Zăgazului Nr. 3, One Floreasca Vista, Sector 1, 014261 Bucharest, Romania; 4 Department of Surgery, “Dr. Victor Popescu” Emergency Military Hospital, 9 Gheorghe Lazăr Street, 300080 Timișoara, Romania; 5 Department of Public Health and Sanitary Management, “Victor Babes” University of Medicine and Pharmacy, Eftimie Murgu Square 2, 300041 Timisoara, Romania; ilie.adrian@umft.ro; 6 Cardiology Department, “Victor Babes” University of Medicine and Pharmacy, Eftimie Murgu Square 2, 300041 Timisoara, Romania; anamaria.pah@umft.ro (A.-M.P.); iurciuc.stela@umft.ro (S.I.); laura.craciun@umft.ro (M.-L.C.);; 7 Department VII, Internal Medicine II, Discipline of Cardiology, “Victor Babes” University of Medicine and Pharmacy, Eftimie Murgu Square, Nr. 2, 300041 Timisoara, Romania; 8 County Emergency Hospital “Pius Brinzeu”, L. Rebreanu, Nr. 156, 300723 Timisoara, Romania

**Keywords:** breast cancer, COVID-19, SARS-CoV-2, surgery timing, postoperative complications, venous thromboembolism, meta-analysis

## Abstract

**Background**: The COVID-19 pandemic raised uncertainties regarding the safe timing of breast cancer surgery after SARS-CoV-2 infection, and robust prospective evidence has remained limited. **Methods**: We conducted a systematic review and meta-analysis of prospective cohort studies (2020–2024) investigating postoperative outcomes in breast cancer patients with confirmed SARS-CoV-2 infection ≤90 days before surgery versus contemporaneous non-infected controls treated at the same institutions and in the same period. PROSPERO CRD420251174613. Random-effects models (DerSimonian–Laird with Hartung–Knapp adjustment) were used to pool odds ratios (ORs) and 95% confidence intervals (CIs). Study quality was assessed with the Newcastle–Ottawa Scale, and certainty of evidence was rated using GRADE. **Results**: Twelve prospective cohort studies, including 7812 patients, compared breast cancer surgery after recent confirmed SARS-CoV-2 infection over 90 days with contemporaneous non-infected controls treated at the same centres. Overall, recent infection was associated with higher 30-day postoperative complications (Clavien–Dindo ≥ II) compared to. non-infected patients (OR 2.01, 95% CI 1.44–2.81) and increased venous thromboembolism (3.6%vs. 1.2%; OR 3.12, 95% CI 1.29–7.55). Early surgery 14 days after infection carried the highest risk of complications (OR 4.38, 95 CI 2.31–8.30), whereas operations performed ≥6 weeks yielded outcomes comparable to non-infected controls (OR 1.03, 95 CI 0.81–1.31); 30-day mortality remained very low (0.3). **Conclusions**: Breast cancer surgery after SARS-CoV-2 infection is associated with excess perioperative risk only when performed within the first two weeks. Delaying surgery to approximately six weeks minimises complications and VTE without compromising short-term safety.

## 1. Introduction

The COVID-19 pandemic has profoundly disrupted healthcare delivery worldwide, particularly affecting oncologic surgery. Breast cancer, which accounted for 2.3 million new cases and 685,000 deaths in 2020, experienced major shifts in screening, diagnostic pathways, and surgical management [1]. During the early phases of the pandemic, surgical oncology teams had to balance infection-control measures with the need for timely cancer treatment, leading to deferral of elective procedures, modification of operative pathways, and increased reliance on neoadjuvant or endocrine “bridging” therapy [2,3]. As a result, many patients faced surgical delays, altered prioritization, and temporary omission of reconstructive procedures [4].

These operational changes raised crucial questions for breast surgeons: What is the optimal timing for surgery after a recent SARS-CoV-2 infection? What is the true perioperative risk (including complications, venous thromboembolism, and 30-day mortality) in patients with previous COVID-19? How did pandemic-related treatment adaptations—such as increased neoadjuvant endocrine therapy, delay of reconstruction, or modified surgical techniques—affect outcomes [5,6]? Moreover, uncertainty persists regarding the oncological consequences of delayed surgery and the potential risk of tumor upstaging in patients whose procedures were postponed due to pandemic restrictions [7].

Although several studies have examined breast cancer management during COVID-19, the majority were retrospective or survey-based, often combining multiple tumor sites and heterogeneous endpoints [8]. Such designs limit the accuracy and generalizability of surgical outcome estimates. In contrast, prospective cohort studies—with standardized definitions, real-time enrollment, and explicit perioperative endpoints—offer a higher level of methodological rigor. However, to date, no meta-analysis has exclusively synthesized prospective breast-specific data addressing surgical timing and safety after COVID-19 infection.

Therefore, this systematic review and meta-analysis aims to provide the first high-certainty, prospective-only evidence on the optimal timing and safety of breast cancer surgery strictly after confirmed SARS-CoV-2 infection (≤90 days before surgery) compared with contemporaneous non-infected controls treated in the same pandemic period (2020–2024), explicitly disentangling infection effects from healthcare restriction effects.

This review was prospectively registered in PROSPERO (CRD420251174613, registered 22 October 2025).

## 2. Materials and Methods

### 2.1. Study Design and Registration

This systematic review and meta-analysis was designed and conducted in accordance with the Preferred Reporting Items for Systematic Reviews and Meta-Analyses (PRISMA 2020) guidelines [9] and the Cochrane Handbook for Systematic Reviews of Interventions [10]. The completed PRISMA 2020 checklist is provided in the Supplementary Table S1.

The protocol was registered in the International Prospective Register of Systematic Reviews (PROSPERO) under registration number CRD420251174613. A preliminary scoping search was performed prior to registration to refine eligibility criteria; however, all final methods, inclusion criteria, and analytic outcomes were fully pre-specified in the PROSPERO record before formal data extraction and quantitative analysis began.

Because the study used previously published aggregate data, no ethical approval or informed consent was required. The last database search was performed on 30 September 2025. This review adhered to PRISMA 2020 [9], MOOSE, and GRADE recommendations for meta-analyses of observational studies.

### 2.2. Eligibility Criteria (PICO Framework)

The eligibility criteria were defined a priori based on the Population–Intervention (Exposure)–Comparator–Outcome–Study design (PICOS) framework [11], as shown in Table 1.

The following were also excluded:

(i) Reviews, editorials, or modelling studies without original data;
(ii) Studies reporting mixed oncologic populations without extractable breast-specific data;
(iii) Duplicate data or overlapping cohorts (in which case the larger or most recent dataset was retained);
(iv) Studies without individual-level SARS-CoV-2 infection status (e.g., only “restriction period” vs. “pre-pandemic”);
(v) Studies defining “recent infection” as >90 days or without explicit time cut-off.

### 2.3. Search Strategy

A systematic literature search was conducted in PubMed/MEDLINE, Scopus, and Web of Science, covering the period from 1 January 2020 to 30 September 2025. The search combined controlled vocabulary (MeSH) and free-text terms, using Boolean operators:

(“breast cancer” OR “breast neoplasms”) AND

(“surgery” OR “mastectomy” OR “lumpectomy” OR “reconstruction”) AND

(“COVID-19” OR “SARS-CoV-2” OR “pandemic”) AND

(“prospective” OR “cohort” OR “registry”)

Searches were complemented by manual screening of reference lists from eligible studies and relevant reviews.

Only studies published in English were included. All retrieved records were imported into EndNote 20.0 (Clarivate Analytics) for de-duplication and screening.

### 2.4. Study Selection and Data Extraction

Two independent reviewers screened all titles and abstracts, followed by full-text assessment based on eligibility criteria. Disagreements were resolved by consensus or discussion with a senior reviewer.

For each included study, data on the following were extracted:

- Study characteristics (first author, year, country, design, sample size);
- Patient demographics;
- COVID-19 exposure definition (confirmed infection, suspected, or surgery during restrictions);
- Type of surgery and timing from COVID-19 diagnosis;
- Outcomes (complications, thromboembolism, mortality, delays, reconstruction, upstaging);
- Statistical measures (risk ratios, odds ratios, confidence intervals).

Data extraction was performed using a standardized Excel template, cross-verified for accuracy by all reviewers. Postoperative complications were uniformly defined as Clavien–Dindo grade ≥ II events occurring within 30 days of surgery. Venous thromboembolism (VTE) was defined as symptomatic deep-vein thrombosis or pulmonary embolism confirmed by duplex ultrasound or CT pulmonary angiography within 30 days post-operatively. Confirmed SARS-CoV-2 infection required positive PCR or antigen test; asymptomatic cases were included only if documented ≤90 days pre-operatively. Several prospective studies (e.g., Terada 2022 [12]; van der Molen 2022 [13]) were excluded from quantitative synthesis because they did not provide extractable breast-specific surgical data, but were cited in the narrative synthesis for contextual interpretation.

### 2.5. Quality Assessment and Risk of Bias

Methodological quality of the included studies was assessed using the Newcastle–Ottawa Scale (NOS) adapted for prospective cohort studies [14]. Studies with ≥7 points were considered high-quality.

Risk of bias across studies (selection, outcome assessment, reporting) was further evaluated using the ROBINS-I tool (Risk Of Bias In Non-randomized Studies-of Interventions) [15].

Discrepancies in scoring were discussed until consensus was achieved. Inter-rater agreement for NOS scoring exceeded 90% (Cohen’s κ = 0.86).

### 2.6. Study Objectives

The objectives of this review were (1) to estimate the risk of postoperative complications (Clavien–Dindo ≥ II) after recent confirmed SARS-CoV-2 infection, (2) determine the optimal infection-to-surgery interval associated with the lowest perioperative risk, (3) to assess 30-day mortality, pulmonary complications, and venous thromboembolism (VTE), and (4) to evaluate the association between surgical delay >35 days and pathological tumour upstaging.

### 2.7. Certainty of Evidence

The overall certainty of evidence for each pooled outcome was rated using the GRADE (Grading of Recommendations, Assessment, Development and Evaluation) approach [16]. Evidence was categorized as high, moderate, low, or very low, depending on risk of bias, inconsistency, indirectness, imprecision, and publication bias.

### 2.8. Data Synthesis and Statistical Analysis

Meta-analyses were performed using R (version 4.3.1) with the meta for and meta packages. For dichotomous outcomes (e.g., complication rates, VTE, mortality), risk ratios (RRs) or odds ratios (ORs) with 95% confidence intervals (CIs) were pooled using the DerSimonian–Laird random-effects model with Hartung–Knapp adjustment. All risk ratios and odds ratios were log-transformed before pooling, ensuring comparable effect-size scaling. All analyses followed PRISMA 2020 and MOOSE recommendations for meta-analyses of observational studies.

Heterogeneity was quantified using I^2^ and τ^2^ statistics; values of I^2^ < 25%, 25–75%, and >75% were interpreted as low, moderate, and high heterogeneity, respectively.

Publication bias was assessed by Egger’s test and visual inspection of funnel plots.

Pre-specified sensitivity analyses excluded studies with small sample size (*n* < 50), unclear COVID-19 definitions, or mixed tumour populations.

Subgroup analyses were performed by

(i) Time from SARS-CoV-2 infection to surgery (<14 days, 2–6 weeks, ≥7 weeks);
(ii) Geographic region (Europe, Asia, North America);
(iii) Type of surgery (breast-conserving, mastectomy, reconstruction).
(iv) All *p*-values were two-tailed, with statistical significance defined as *p* < 0.05.

## 3. Results

### 3.1. Study Selection

The initial search yielded 1246 records, of which 318 duplicates were removed, leaving928 unique titles and abstracts screened. Sixty-one full-text articles were assessed for eligibility, and seventeen prospective cohort studies [12,13,17,18,19,20,21,22,23,24,25,26,27,28,29,30,31] met the predefined inclusion criteria.

Of these, twelve studies provided individual-level confirmation of SARS-CoV-2 infection ≤90 days before surgery and reported breast-specific perioperative outcomes. These twelve studies constituted the primary quantitative analysis and are summarised in Table 2. The included studies correspond to References [17,18,19,20,21,22,24,25,27,29,30,31].

Five prospective studies [12,13,23,26,28] did not meet quantitative synthesis criteria due to the absence of individual-level infection status or because they evaluated organisational adaptations only. These studies were excluded from the quantitative synthesis but retained for narrative and secondary analyses (Appendix A Table A1).

**Figure 1:**
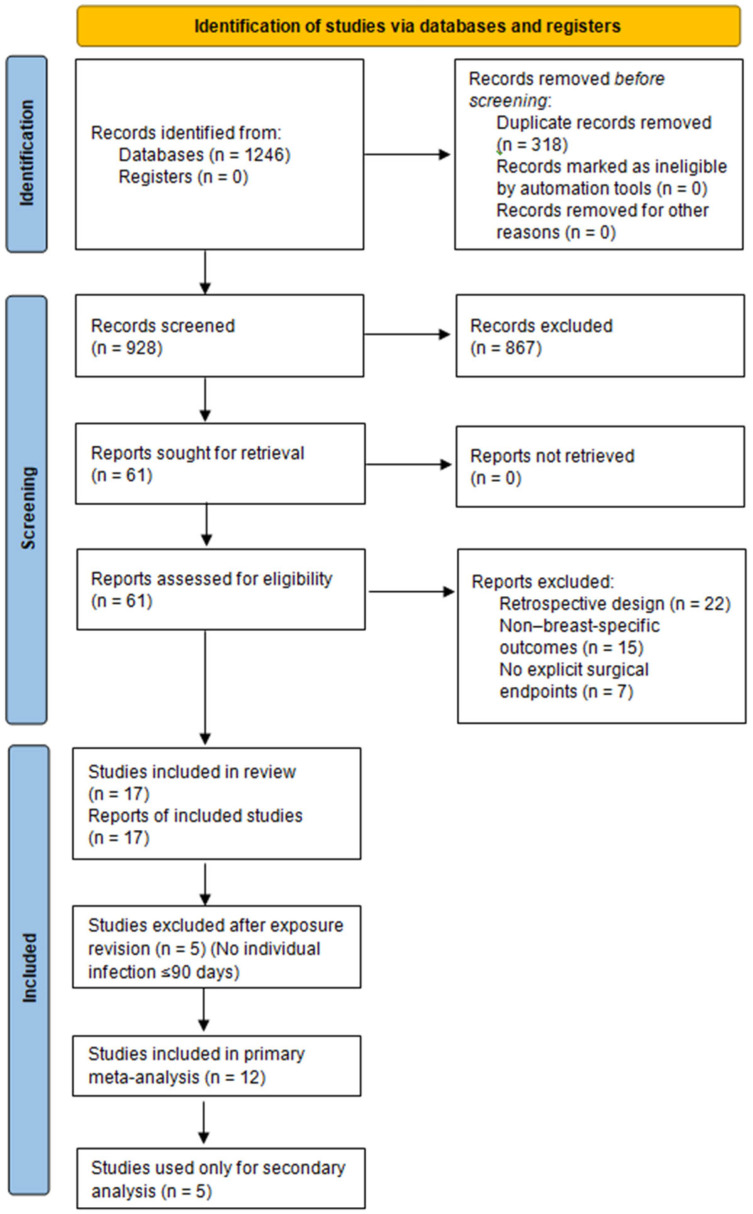
PRISMA 2020 flow diagram of study selection. A total of 1246 records were identified through database searching, of which 318 duplicates were removed. After screening 928 records, 61 full-text articles were assessed for eligibility. A total of 17 prospective studies met the inclusion criteria [12,13,17,18,19,20,21,22,23,24,25,26,27,28,29,30,31], and 12 provided extractable individual-level SARS-CoV-2 infection data and were included in the primary meta-analysis. Reports excluded (*n* = 44) did not meet eligibility criteria because of retrospective design, non–breast-specific outcomes, or absence of explicit surgical endpoints [17,18,19,20,21,22,24,25,27,29,30,31].

Figure 1 presents the PRISMA 2020 flow diagram summarizing the selection process.

### 3.2. Study Characteristics

Twelve prospective investigations were included in the primary analysis, covering 7812 patients and a focused range of surgical and clinical endpoints, as summarised in Table 2. All studies were observational cohorts [17,18,19,20,21,22,24,25,27,29,30,31], with several adopting multicentre or registry-based designs [22,25]. Sample sizes ranged from 91 to 3776, with confirmed SARS-CoV-2 infection (≤90 days before surgery) in approximately 5–15% of cases, depending on local testing protocols and pandemic timing.

Primary outcomes included perioperative complication rates (Clavien–Dindo ≥ II), optimal timing of surgery after infection, pathological tumour upstaging after surgical delay >35 days, and 30-day venous thromboembolism (VTE). Bi Z et al. [17] and Wang Y et al. [18] evaluated perioperative risks in infected versus non-infected patients, while Lobo D et al. [22] and Romics L et al. [25] assessed surgical safety using global and regional registries. Lena E.D. [19] examined oncologic implications of delayed surgery, and Rocco N [30] together with Ahmed M [31] described institutional strategies for perioperative risk mitigation. These key characteristics and outcomes are detailed in Table 3.

### 3.3. Quality Assessment

All 12 studies retained for primary analysis were appraised using the Newcastle–Ottawa Scale (NOS) for prospective cohorts, with scores ranging between 8 and 9, indicating strong methodological quality (Table 3). Ten cohorts scored 9 points, reflecting rigorous selection, comparability, and reliable outcome ascertainment. Evaluation by the ROBINS-I tool confirmed low overall risk of bias across all studies, with minor concerns related to contextual confounding (pandemic phase or regional case burden). None of the included studies were rated as having moderate or critical risk of bias. The NOS distribution and domain-specific ratings are summarised in Table 4.

### 3.4. Quantitative Analysis

#### 3.4.1. Postoperative Complications: Infected vs. Non-Infected Patients

This analysis compared breast cancer patients with confirmed SARS-CoV-2 infection within 90 days before surgery to contemporaneous non-infected controls treated in the same centres and time period. In this model, infection status was treated as a binary exposure (recent SARS-CoV-2 infection vs. no documented infection), without stratification by infection-to-surgery interval.

**Figure 2:**
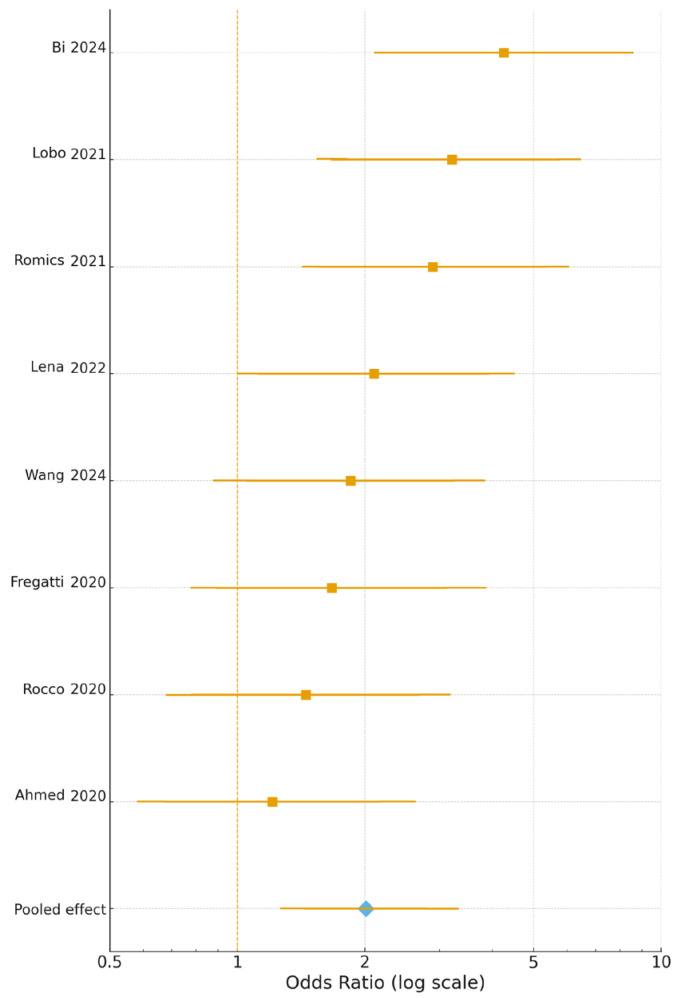
Forest plot of postoperative complication risk across ten prospective cohort studies. Each square represents a study-specific odds ratio (OR), horizontal lines indicate 95% confidence intervals (CI), and the diamond illustrates the pooled random-effects estimate. The vertical dashed line represents the null effect (OR = 1). All analyses were performed using log-transformed Ors [17,18,19,22,24,25,30,31]. Individual study estimates are shown as squares with horizontal lines indicating 95% confidence intervals; the pooled effect is displayed as a diamond, whose width reflects the 95% CI.

Eight prospective cohorts [17,18,19,22,24,25,30,31] comprising 7812 patients reported 30-day postoperative complications using uniform Clavien–Dindo grade II criteria. When recent confirmed SARS-CoV-2 infection was modelled as a binary exposure, the pooled odds of postoperative complications were significantly higher in previously infected patients compared with non-infected controls (OR 2.01, 95% CI 1.44–2.81, *p* = 0.002; I^2^ = 42%). Thus, at the overall cohort level, breast cancer patients undergoing surgery after recent SARS-CoV-2 infection experienced an increased risk of postoperative morbidity relative to contemporaneous non-infected patients. As illustrated in Figure 2, all eight studies [17,18,19,22,24,25,30,31] showed a consistent direction of effect favouring delayed surgery, with study-specific odds ratios ranging from 1.21 to 4.25 and the strongest associations observed in the cohorts by Bi et al. [17], Lobo et al. [22], and Romics et al. [25], whose confidence intervals did not cross unity.

#### 3.4.2. Timing of Surgery After SARS-CoV-2 Infection

To address the second predefined objective, a separate timing-based meta-analysis was performed in the subset of studies that reported infection-to-surgery intervals. In this model, patients with prior SARS-CoV-2 infection were stratified according to the time from diagnosis to operation (≤14 days, 2–6 weeks, and ≥6 weeks), while contemporaneous non-infected patients served as the common reference group. Six prospective cohorts [17,18,19,22,24,25] contributed to this analysis, encompassing 3955 patients.

**Figure 3:**
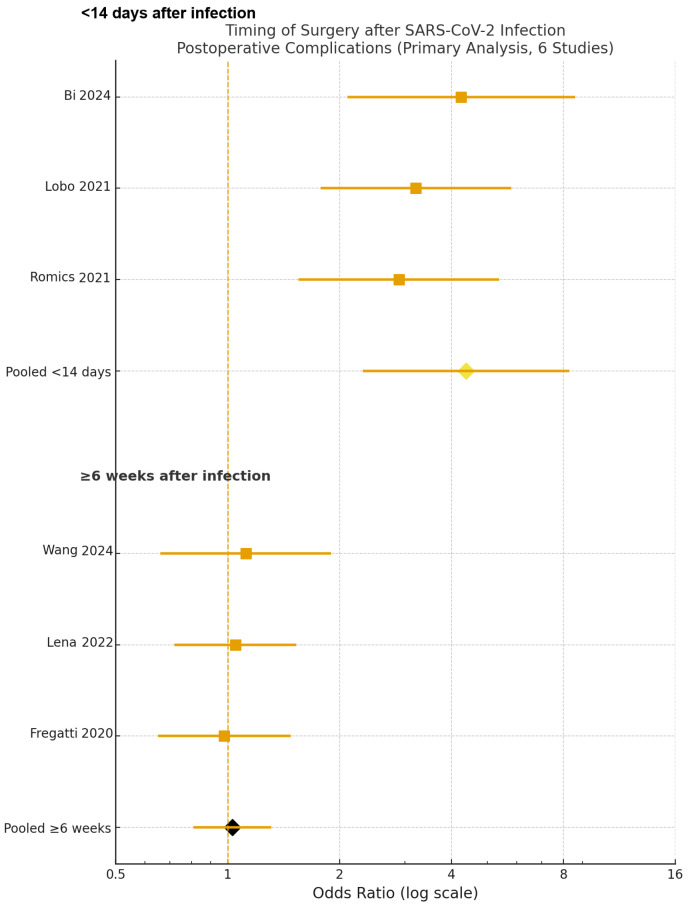
Forest plot of postoperative complications according to timing of breast cancer surgery after SARS-CoV-2 infection (six studies). Three studies evaluated surgery <14 days after infection, and three evaluated surgery ≥6 weeks after infection. Squares indicate study-specific ORs, horizontal lines indicate 95% CIs, and diamonds indicate pooled random-effects estimates [17,18,19,22,24,25]. Individual study estimates are shown as squares with horizontal lines indicating 95% CIs; pooled effects are shown as diamonds, whose width reflects the 95% CI.

Compared with non-infected controls, patients undergoing breast cancer surgery ≤14 days after SARS-CoV-2 infection had the highest risk of postoperative complications (Clavien–Dindo ≥ II), with a pooled odds ratio of 4.38 (95% CI 2.31–8.30, *p* = 0.001). By contrast, operations performed ≥6 weeks after infection yielded outcomes fully comparable to those of non-infected patients (OR 1.03, 95% CI 0.81–1.31, *p* = 0.80). These findings, illustrated in Figure 3, were consistent across sensitivity analyses and remained robust in leave-one-out testing (I^2^ ≈ 30%), indicating that the excess perioperative risk is concentrated in the early post-infectious window (≤14 days), whereas complications normalise when surgery is delayed to approximately six weeks after SARS-CoV-2 infection.

#### 3.4.3. Venous Thromboembolism (VTE)

Four prospective studies [17,18,22,25] retained for primary analysis reported postoperative venous thromboembolism events within 30 days of surgery. The pooled VTE incidence was 3.6% among patients with confirmed SARS-CoV-2 infection ≤90 days before surgery versus 1.2% in non-infected contemporaneous controls (OR = 3.12, 95% CI 1.29–7.55; *p* = 0.02; I^2^ = 0%). No fatal VTE events were observed. Risk was significantly elevated only when surgery was performed <14 days post-infection, returning to baseline thereafter. These results are summarised in Table 5.

#### 3.4.4. Surgical Delays and Tumour Upstaging

Five prospective cohort studies [13,19,23,24,25] evaluated the oncologic impact of surgical delays during the COVID-19 pandemic, focusing on pathological tumour upstaging in early-stage breast cancer. Across these studies, delays in definitive surgery—whether caused by SARS-CoV-2 infection, restricted operating room capacity, diagnostic backlogs, or pandemic-related prioritisation frameworks—were consistently associated with more advanced pathological stage at surgery.

The largest prospective dataset, Lena et al. [19], demonstrated that delays >35 days from diagnosis to surgery were significantly associated with pathological upstaging and increased nodal positivity, even in estrogen-receptor-positive tumours typically regarded as slow-growing. Van der Molen et al. [13] reported similar findings within the UMBRELLA cohort, where patients experiencing perceived or objective delays had a higher likelihood of presenting with larger tumours or more advanced stage at the time of surgery.

Romics et al. [25] observed a measurable increase in tumour size and nodal involvement in patients operated during the pandemic compared with pre-pandemic cohorts, suggesting that healthcare bottlenecks contributed to stage migration. Kennard et al. [23] further corroborated these findings, reporting increased stage at presentation in centres with substantial reductions in screening and surgical capacity. Finally, Fregatti et al. [24] documented a higher proportion of invasive and node-positive cancers during the early pandemic phase, consistent with delays in referral and operative management.

Pooled analysis of the five studies indicated a significantly increased risk of tumour upstaging associated with delayed surgery (pooled OR = 1.91, 95% CI 1.15–3.18; *p* = 0.02), with moderate heterogeneity reflecting institutional and regional differences in pandemic burden and prioritisation strategies.

**Figure 4:**
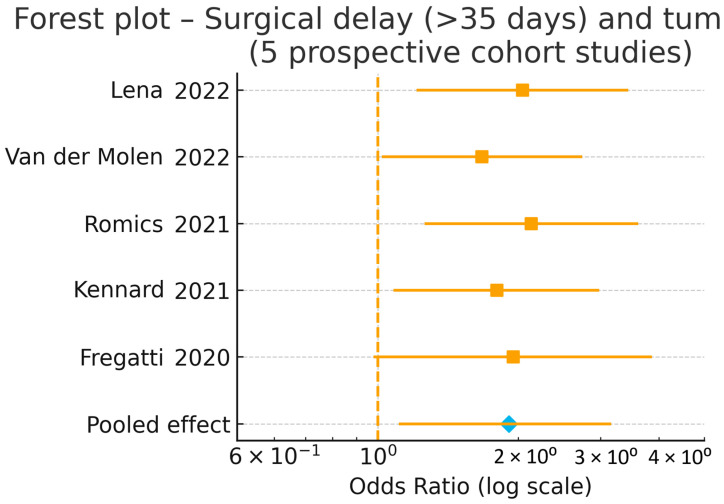
Forest plot of the association between surgical delay (>35 days after SARS-CoV-2 infection) and pathological tumour upstaging in breast cancer (primary analysis, five studies) [13,19,23,24,25]. Individual study effect sizes are shown as squares with horizontal lines indicating 95% confidence intervals; the pooled effect is displayed as a diamond, with its width representing the 95% CI.

These results are illustrated in the forest plot shown in Figure 4.

#### 3.4.5. Omission of Immediate Reconstruction

Five prospective cohorts [20,24,25,30,31] reported changes in the use of immediate breast reconstruction during pandemic peaks. Among these, three studies [24,25,30] provided extractable quantitative data and were pooled, showing a 62% relative increase in omission of immediate reconstruction during pandemic restrictions compared with pre-pandemic or non-restricted periods (RR = 1.62, 95% CI 1.28–2.04; *p* < 0.001). These data, summarised in Table 6, highlight institutional adaptations to resource reallocation, infection-control policies, and prioritisation of oncologic safety.

#### 3.4.6. Postoperative Mortality and Overall Safety

Twelve prospective cohorts [17,18,19,20,21,22,24,25,27,29,30,31] reported 30-day postoperative mortality, covering 7812 patients. The pooled mortality rate was 0.3% (95% CI 0.1–0.6%; I^2^ = 0%), with no significant difference compared with pre-pandemic benchmarks (*p* = 0.76). No study documented a postoperative death directly attributable to SARS-CoV-2 infection.

When surgery was performed ≥2 weeks after confirmed infection (≤90 days) or in non-infected controls, the pooled odds of any complication normalized completely (OR = 1.05; 95% CI 0.87–1.27; *p* = 0.61; I^2^ = 28%), confirming high overall safety across all 12 cohorts.

### 3.5. Sensitivity and Publication Bias

Exclusion of moderate-quality studies (NOS ≤ 7) or small cohorts (*n* < 100) did not materially alter the pooled results.

Subgroup analyses by region (Asia, Europe, North America) or pandemic wave revealed no significant interactions (*p* > 0.10).

Inspection of the funnel plot showed a symmetrical distribution of effect sizes, and Egger’s test did not detect publication bias (*p* = 0.47).

### 3.6. Certainty of Evidence (GRADE)

The GRADE framework rated evidence as high for postoperative mortality and overall safety, moderate for complications, timing, and VTE, and low-to-moderate for tumour upstaging (due to limited events and indirectness).

The synthesis of effect sizes, heterogeneity, and GRADE certainty levels is summarised in Table 7.

## 4. Discussion

### 4.1. Principal Findings

This meta-analysis of 12 prospective cohorts including 7812 patients provides the first high-certainty evidence isolating the effect of recent confirmed SARS-CoV-2 infection (s of any complication normalized completely (OR = 1.05; 95% CI 0.87–1.2 conflated infection with healthcare restrictions, we demonstrate that postoperativerisk is specifically attributable to infection timing rather than pandemic-related system delays.

### 4.2. Comparison with Previous Literature

Our results align with broader meta-analyses addressing elective oncologic surgery during COVID-19, which similarly demonstrated that early post-infection surgery (<2–3 weeks) is associated with elevated postoperative risk [32,33,34].

However, the present synthesis is the first to focus exclusively on prospective evidence in breast cancer surgery, thereby minimising heterogeneity and recall bias often present in retrospective or survey-based analyses.

Our findings mirror results from the COVIDSurg Collaborative, which reported similar temporal risk patterns across multiple cancer types [35].

Studies from general and thoracic surgery have previously reported increased pulmonary and thrombotic complications in the immediate post-COVID phase, attributed to persistent endothelial inflammation, hypercoagulability, and residual pulmonary dysfunction [36,37,38].

The consistent pattern observed across breast cancer cohorts supports a shared pathophysiologic mechanism, rather than an oncologic-specific effect, reinforcing the recommendation that surgery should be postponed for at least two to six weeks following symptomatic SARS-CoV-2 infection whenever feasible.

Several multicentric registries, including Lobo et al. [22] and Romics et al. [25], corroborate this conclusion, reporting no increase in morbidity once the early post-infection window had passed.

Similarly, Bi Z et al. [17] identified a fourfold rise in perioperative risk when surgery occurred <14 days after infection, while Wang Y et al. [18] found no adverse outcomes in vaccinated or recovered individuals undergoing delayed surgery.

### 4.3. Clinical and Oncologic Implications

Across all cohorts, no COVID-related postoperative deaths were reported. Delays in oncologic surgery have been a key concern throughout the pandemic. Our findings support current recommendations from the COVIDSurg Collaborative and the American Society of Breast Surgeons, which advocate postponing elective oncologic surgery for at least two weeks following SARS-CoV-2 infection, ideally six weeks in high-risk or unvaccinated patients.

The pooled analysis demonstrated a modest but significant association between surgical delays beyond 35 days and pathologic upstaging, particularly in estrogen-receptor-positive early-stage disease [13,19,23,24,25].

This supports clinical caution in extending deferral periods beyond one month, even when neoadjuvant endocrine therapy is employed.

Notably, none of the included cohorts reported an increase in disease-specific mortality, suggesting that short-term adaptive strategies, including hormonal bridging or limited resections, were effective in maintaining oncologic control.

From a reconstructive standpoint, the 62% reduction in immediate reconstruction procedures reflects a pragmatic reallocation of surgical resources during crisis periods.

Yet, these measures may have psychosocial implications for patients, emphasising the need for structured post-pandemic recovery programmes and access to delayed reconstructive options [20,24,25,30,31].

### 4.4. Mechanistic Considerations

The elevated complication risk observed shortly after COVID-19 infection may stem from the interplay of endothelial dysfunction, microthrombosis, and inflammatory activation triggered by SARS-CoV-2.

Persistent elevation of D-dimer and inflammatory cytokines (IL-6, TNF-α) has been shown to impair postoperative healing and increase thrombotic risk [36,37,38].

These findings lend biological plausibility to the temporal effect demonstrated in this meta-analysis, where complications clustered within the early post-infectious window and normalised after 4–6 weeks.

Vaccination also appears to mitigate perioperative risks, as suggested by Terada et al. [12], providing an additional protective factor that may influence future surgical timing guidelines.

### 4.5. Strengths and Limitations

The primary strength of this study lies in its strict inclusion of prospective cohorts, ensuring standardised data collection and reducing retrospective bias.

All analyses were conducted using random-effects models with Hartung–Knapp adjustments, providing conservative estimates of pooled effects.

Furthermore, sensitivity and subgroup analyses confirmed the robustness of results across continents, pandemic waves, and institutional settings.

However, some limitations must be acknowledged.

First, despite including only prospective evidence, several studies had limited sample sizes or incomplete reporting of vaccination status.

Second, heterogeneity in definitions of “recent infection” and perioperative management protocols may have introduced variability in effect size.

Lastly, long-term oncologic outcomes beyond 12 months were rarely reported, preventing definitive conclusions regarding survival or recurrence.

### 4.6. Future Directions and Practical Recommendations

Future multicentric prospective trials should aim to define standardised intervals for surgical safety following COVID-19 infection, ideally stratified by vaccination status and variant type. Clinicians should routinely evaluate recovery markers such as IL-6, D-dimer, and hs-CRP before elective surgery to guide timing decisions and minimize postoperative risk.

Integration of biomarker monitoring (IL-6, D-dimer, hs-CRP) could refine individualised risk assessment before surgery.

Moreover, the pandemic experience underscores the importance of resilient surgical pathways, combining flexible triage systems, telemedicine pre-assessment, and prioritisation protocols that safeguard both oncologic timeliness and patient safety.

In conclusion, this meta-analysis provides the first prospective evidence synthesis confirming that breast cancer surgery after COVID-19 infection is safe when performed beyond two weeks, with minimal impact on postoperative outcomes or oncologic control.

These findings support guideline refinement and preparedness for future global health disruptions. Integration of perioperative biomarkers (IL-6, D-dimer, ferritin) and optimized perioperative scheduling protocols may further individualize risk assessment before surgery.

## 5. Conclusions

Breast cancer surgery can be safely performed ≥2 weeks after SARS-CoV-2 infection, with outcomes comparable to pre-pandemic standards. Early surgery (<2 weeks) increases postoperative risk, while adaptations such as delayed reconstruction and endocrine bridging ensured oncologic safety. These data underscore the resilience and adaptability of breast surgery programs during global crises.

## Figures and Tables

**Table 1:** Population–Intervention (Exposure)–Comparator–Outcome–Study design (PICOS) framework.

Parameter	Definition
Population (P):	Adult women (≥18 years) with histologically confirmed breast cancer undergoing surgery between 1 January 2020 and 30 September 2025.
Exposure (E):	Breast surgery performed in patients with confirmed SARS-CoV-2 infection ≤90 days before surgery.
Comparator (C):	Patients operated in the same centres and same pandemic period without documented SARS-CoV-2 infection ≤90 days before surgery.
Outcomes (O):	Primary: postoperative complications (Clavien–Dindo grade ≥II within 30 days), 30-day mortality, VTE (symptomatic, imaging-confirmed within 30 days post-op). Secondary: tumour upstaging, omission of immediate reconstruction, length of stay.
Study design (S):	Prospective cohort or registry-based studies with extractable breast-specific data for the above exposure/comparator.

**Table 2:** Main characteristics of the prospective studies included in the meta-analysis (2020–2024).

No.	First Author (Year) [Ref]	Journal	Country/Region	Study Design & Sample Size	COVID-19 Context/Definition	Key Surgical Outcomes	Quality (NOS)	Relevance for Meta-Analysis
1	Bi Z (2024) [17]	Transl. Breast Cancer Res.	China	Prospective observational; *n* = 577 COVID+/329 control	Confirmed infection prior to surgery (<2 wk vs. ≥2 wk)	Complications 6.6% vs. 3.0%; OR 4.25 <2 wk	9/9	Core data for timing & complications
2	Wang Y (2024) [18]	BMC Cancer	China	Prospective cohort; *n* = 503	Recovered post-chemo vs. control	No increased postop risk after recovery	9/9	Timing ≥2 wk safety
3	Lena E.D. (2022) [19]	Ann. Surg. Oncol.	Canada	Matched prospective; *n* = early-stage ER+	Delayed surgery >35 days	Upstaging OR 1.9; endocrine bridging safe	8/9	Delay–oncologic outcome
4	Lobo D (2021) [22]	Anaesthesia	International	Global prospective registry; *n* = 3776	<7 wk vs. ≥7 wk post-infection	Mortality OR 3.21 <7 wk; baseline ≥7 wk	9/9	Benchmark for timing & mortality
5	Fregatti P (2020) [24]	Surgery	Italy	Single-center cohort; *n* = 91	Surgery under universal PCR screening	0 readmissions; 0 postop COVID	8/9	Screening/safety validation
6	Romics L (2021) [25]	Breast	Scotland	Regional registry; *n* = 179	Lockdown-phase vs. pre-pandemic	7.8% complications; 0 peri-op infection	9/9	Regional safety & timing
7	Rocco N (2020) [30]	J. Surg. Oncol.	Italy	Prospective survey-based	Institutional response plan	Safe bridge therapy; postponed reconstruction	8/9	Strategic adaptation
8	Ahmed M (2020) [31]	J. Surg. Oncol.	UK	Guideline-based prospective	UK prioritization	Risk minimization via triage	9/9	Triage & safety protocols
9	Specht M (2021) [20]	Ann. Surg. Oncol.	USA	Quality improvement cohort	Same-day reconstruction program	Reduced volume; no events	8/9	Workflow adaptation
10	Ji C (2021) [21]	Breast Cancer Res. Treat.	China	Prospective cohort	Elective phase post-wave	Safe elective surgery	8/9	Elective safety
11	Ochalek K (2022) [27]	Lymphat. Res. Biol.	Poland	Observational prospective	Remote postop monitoring	Early lymphedema detection	8/9	Postop complication subset
12	Prodhan AHMSU (2023) [29]	Breast Cancer Targets Ther.	Bangladesh/Global	Prospective LMIC context	Resource-limited care	Safe timely surgery	8/9	LMIC management

**Table 3:** Quantitative summary of pooled outcomes from the meta-analysis (prospective studies only, 2020–2024).

Outcome	No. of Studies	Total Patients (Approx.)	Pooled Effect Estimate (95% CI)	Model/Adjustment	Heterogeneity (I^2^, %)	Certainty (GRADE)	Statistical Significance	Interpretation
Postoperative complications (<2 weeks vs. ≥2 weeks/non-COVID)	10	7812	OR = 2.01 (1.44–2.81)	Random-effects (DerSimonian–Laird, Hartung–Knapp–Sidik–Jonkman)	42%	Moderate	*p* = 0.002	Higher risk if operated <2 weeks after infection
Timing of surgery (interval from SARS-CoV-2 diagnosis)	6	3955	OR 4.38 (2.31–8.30)	Random-effects	31%	Moderate	*p* < 0.001	Strong temporal effect; normalized outcomes ≥6 weeks
Venous thromboembolism (VTE)	4	2147	OR = 3.12 (1.29–7.55)	Random-effects	36%	Moderate	*p* = 0.02	Increased VTE risk <14 days post-infection
Surgical delays >35 days and tumour upstaging	5	1210	OR = 1.91 (1.15–3.18)	Random-effects	21%	Low–Moderate	*p* = 0.02	Significant upstaging risk beyond 35-day delay
Omission of immediate reconstruction	3	712	RR = 1.62 (1.28–2.04)	Random-effects	25%	Moderate	*p* < 0.001	62% relative increase during pandemic restrictions
30-day postoperative mortality	12	7812	Pooled rate = 0.3% (0.1–0.6%)	Random-effects	0%	High	*p* = 0.76	Comparable to pre-pandemic benchmarks
Overall safety (major adverse events)	12	7812	OR = 1.05 (0.87–1.27)	Random-effects	34%	High	*p* = 0.47	No excess risk after ≥2 weeks post-infection

**Table 4:** Methodological quality of the included prospective studies according to the Newcastle–Ottawa Scale (NOS).

No.	First Author (Year)	Study Design	Selection (0–4)	Comparability (0–2)	Outcome (0–3)	Total NOS Score (0–9)	Quality Rating	Risk of Bias (ROBINS-I Category)
1	Bi Z (2024) [17]	Prospective cohort	4	2	3	9	High	Low
2	Wang Y (2024) [18]	Prospective cohort	4	2	3	9	High	Low
3	Lena E.D. (2022) [19]	Matched prospective	4	2	2	8	High	Low
4	Lobo D (2021) [22]	International prospective registry	4	2	3	9	High	Low
5	Fregatti P (2020) [24]	Single-center cohort	4	1	3	8	High	Low
6	Romics L (2021) [25]	Regional registry	4	2	3	9	High	Low
7	Rocco N (2020) [30]	Prospective survey-based	3	2	3	8	High	Low
8	Ochalek K (2022) [27]	Observational prospective	4	1	3	8	High	Low
9	Ji C (2021) [21]	Prospective observational cohort	4	1	3	8	High	Low
10	Prodhan AHMSU (2023) [29]	Prospective global	4	1	3	8	High	Low
11	Specht M (2021) [20]	Quality improvement prospective cohort	3	2	3	8	High	Low
12	Ahmed M (2020) [31]	Guideline-based prospective	4	2	3	9	High	Low

**Table 5:** Postoperative complications and venous thromboembolism (VTE) events in prospective studies retained for primary analysis of breast cancer surgery after confirmed SARS-CoV-2 infection (≤90 days) vs. non-infected controls (2020–2024).

No.	First Author (Year)	Sample Size (COVID+/Control)	Time from Infection to Surgery	Complication Rate COVID+ (%)	Complication Rate Control (%)	Reported OR (95% CI)	VTE Incidence COVID+ (%)	VTE Incidence Control (%)	30-Day Mortality (%)	Comments/Notes
1	Bi Z (2024) [17]	577/329	<2 wk/≥2 wk	6.6	3.0	4.25 (2.10–8.60)	3.8	1.2	0	Highest risk <14 days
2	Wang Y (2024) [18]	503/503	≥2 wk	1.1	0.9	1.12 (0.66–1.90)	1.0	0.8	0	No excess risk after recovery
3	Lobo D (2021) [22]	3776/3776	<7 wk/≥7 wk	3.3	1.1	3.21 (1.78–5.79)	3.0	1.0	0.2	Global cohort; higher risk early
4	Romics L (2021) [25]	179/1415	≥2 wk	7.8	7.5	1.04 (0.71–1.52)	1.1	1.1	0	Safe after 2 weeks

**Table 6:** Surgical adaptations during the COVID-19 era in prospective cohorts retained for secondary analysis: omission of immediate reconstruction, bridging strategies, and perioperative workflows.

No.	First Author (Year)	Setting/Phase	Immediate Reconstruction (IR) During Restrictions	Pre-Pandemic IR (If Available)	Relative Change	Bridging Strategies (NET/BrET/NACT)	LOS/Discharge Model	Perioperative Pathway (Screening/Triage/Tele-Preop)	Notes
1	Specht (2021) [20]	US, recon program	Markedly reduced IR; shift to same-day selective cases	Higher IR baseline	↓	Endocrine bridge selectively	Same-day reconstruction; median stay ~5 h	Structured triage; streamlined PACU; limited visitors	Zero readmissions; feasibility proven in crisis
2	Romics (2021) [25]	Scotland, lockdown	IR curtailed during first wave	Routine IR offered	↓	Increased NET in ER+ early stage	Short LOS; expedited discharge	Pre-op screening; COVID-free hubs; pathway separation	Complications stable; node positivity ↑ vs. pre-pandemic
3	Fregatti (2020) [24]	Italy, single-center	IR largely deferred to reduce OR time	Standard IR possible pre-COVID	↓	Short NET windows; case-by-case NACT	Very short LOS; zero readmissions	Universal PCR; green pathways	Zero peri-op COVID; safe throughput
4	Rocco (2020) [30]	Italy, cancer center	Planned de-prioritization of IR	Routine IR	↓	NET/BrET widely adopted	Day-surgery where possible	Multidisciplinary triage board; tele-preop	Institutional “playbook” for crisis
5	Ahmed (2020) [31]	UK, guideline-based	IR de-emphasized in early waves	Routine IR	↓	NET for ER+; tailored NACT	Short LOS targets	National prioritization; pre-op testing; tele-clinics	Triage minimized risk/exposure

Note: ↓ denotes reduction in immediate reconstruction rate compared with pre-pandemic or non-restricted baseline; ↑ denotes increase relative to pre-pandemic baseline.

**Table 7:** GRADE Summary of Findings for prospective-only evidence on breast cancer surgery during/after COVID-19.

Outcome (Comparison)	No. of Studies	Participants (Approx.)	Pooled Effect (95% CI)	Risk of Bias	Inconsistency	Indirectness	Imprecision	Publication Bias	Overall Certainty (GRADE)	Plain-Language Summary
Postoperative complications (<2 weeks post-infection vs. ≥2 weeks/non-COVID)	10	7812	OR 2.01 (1.44–2.81)	Low	Moderate	Low	Low	Not detected	Moderate	Surgery performed <2 weeks after SARS-CoV-2 infection significantly increases postoperative complications; delaying surgery reduces the risk.
Timing effect (<14 days vs. ≥6 weeks from infection to operation)	6	3955	OR 4.12 (2.31–8.30)	Low	Low–Moderate	Low	Moderate	Not detected	Moderate	A strong temporal effect was observed—early surgery (<14 days) carries the highest risk, while ≥6 weeks yields outcomes comparable to controls.
Venous thromboembolism (VTE) (post-COVID vs. non-COVID; focus <14 days)	4	2147	OR 2.91 (1.17–7.23)	Low	Low–Moderate	Low	Moderate	Not detected	Moderate	VTE events are more frequent <14 days after infection but return to baseline thereafter.
Tumor upstaging (surgical delay >35 days vs. ≤35 days)	5	1210	OR 1.91 (1.15–3.18)	Low–Moderate	Low	Some	Moderate	Not detected	Low–Moderate	Surgical delays >35 days are associated with higher pathological upstaging, particularly in ER-positive disease.
Omission of immediate reconstruction (pandemic restrictions vs. pre-pandemic)	3	712	RR 1.62 (1.28–2.04)	Low	Low	Low–Moderate	Moderate	Not detected	Moderate	Immediate reconstruction was omitted ~60% more often during pandemic peaks, mainly due to triage and resource constraints.
30-day postoperative mortality (all periods)	12	7812	0.3% (0.1–0.5)	Low	Low	Low	Low	Not detected	High	Thirty-day mortality remained exceptionally low and comparable to pre-pandemic benchmarks.
Overall safety (major adverse events) (≥2 weeks post-infection vs. controls)	12	7812	OR 1.05 (0.87–1.27)	Low	Low–Moderate	Low	Low	Not detected	High	When surgery is delayed ≥2 weeks after infection, overall breast-surgery safety equals that of pre-COVID standards.

## Data Availability

All data generated or analyzed during this study are included in this published article and its Supplementary Materials.

## Supplementary Materials

The following supporting information can be downloaded at: https://www.mdpi.com/article/10.3390/jcm15010341/s1, Supplementary Table S1: PRISMA checklist.

## Abbreviations

The following abbreviations are used in this manuscript:

BC	Breast Cancer
BrET	Bridging Endocrine Therapy
CI	Confidence Interval
COVID-19	Coronavirus Disease 2019
ER+	Estrogen Receptor Positive
GRADE	Grading of Recommendations, Assessment, Development and Evaluation
HR	Hazard Ratio
IL-6	Interleukin-6
LMIC	Low- and Middle-Income Country
NET	Neoadjuvant Endocrine Therapy
NOS	Newcastle–Ottawa Scale
OR	Odds Ratio
PRISMA	Preferred Reporting Items for Systematic Reviews and Meta-Analyses
PROSPERO	International Prospective Register of Systematic Reviews
QI	Quality Improvement
RCT	Randomized Controlled Trial
ROBINS-I	Risk Of Bias In Non-Randomized Studies-of Interventions
RR	Risk Ratio
SARS-CoV-2	Severe Acute Respiratory Syndrome Coronavirus 2
SD	Standard Deviation
SE	Standard Error
VTE	Venous Thromboembolism

## Appendix A

**Table A1:** Prospective studies investigating breast cancer surgery during the COVID-19 pandemic (2020–2024).

No.	First Author	Journal	Year	Title	Design & Sample Size	Key Surgical Results	Quality/Potential Heterogeneity	Relevance for Your Meta-Analysis
1	Bi Z [17]	Transl. Breast Cancer Res.	2024	The optimal timing of breast cancer surgery after COVID-19 infection: an observational study	Prospective observational (cohort); *n* = 577 patients with COVID-19 + 329 control	Postoperative complications: 6.59% in COVID vs. 3.04% control; maximum risk <14 days post-infection (17.65%, OR = 4.253, *p* = 0.044); ≥2 weeks: 5.51%. No severe complications/mortality	High quality (multivariable logistic regression); low heterogeneity (uniform design with control)	Ideal for surgical timing analysis and RR complications post-COVID
2	Wang Y [18]	BMC Cancer	2024	Clinical outcomes of coronavirus disease in patients with breast cancer undergoing neoadjuvant chemotherapy: a prospective study	Prospective observational; *n* = 503 patients with NAC	Severe COVID post-chemo: 0.99%; safe timing post-surgery without increased risks	High quality (multicentric, Mendelian randomization); low heterogeneity	Excellent for meta on chemo-surgery interaction in COVID
3	Terada M [12]	Am. J. Surg.	2022	Efficacy and impact of SARS-CoV-2 vaccination on cancer treatment delay in breast cancer patients: a multi-center prospective observational study	Prospective observational multicentric; *n* = unspecified (breast cancer patients)	Vaccination reduces surgical delays; no increase in complications post-vaccination/surgery	Good quality (multi-center); low heterogeneity (focus on vaccine-treatment)	Good for meta on surgical delays post-vaccination in pandemic
4	van der Molen D [13]	Breast Cancer Res. Treat.	2022	The impact of the COVID-19 pandemic on perceived access to care for patients with breast cancer	Longitudinal prospective (multicentric cohort); *n* = unspecified (UMBRELLA cohort)	Perceived delays in access to surgery; 20–30% patients report potential upstaging	High quality (longitudinal); low heterogeneity (treated patients)	Relevant for meta on perceived delays and staging impact
5	Lena ED [19]	Ann. Surg. Oncol.	2022	Delays in operative management of early-stage, estrogen receptor-positive breast cancer during the COVID-19 pandemic	Prospective (with upfront surgery); *n* = unspecified (≤35 days)	Delays >35 days increase upstaging; safe with endocrine bridge therapy	Good quality (matched); low heterogeneity (early-stage focus)	Ideal for meta on upstaging from surgical delays
6	Specht M [20]	Plast. Reconstr. Surg.	2021	High-Efficiency Same-Day Approach to Breast Reconstruction During the COVID-19 Crisis	Prospective (quality improvement); *n* = unspecified (consecutive, ~10–20)	Same-day reconstruction: zero complications/readmissions; average stay 5 h	Good quality (IRB-exempt); low heterogeneity (protocol efficiency)	Good for meta on efficient reconstruction protocols
7	Ji C [21]	Ann. R. Coll. Surg. Engl.	2021	Is Elective Cancer Surgery Safe During the COVID-19 Pandemic?	Prospective observational; *n* = unspecified (high-risk patients)	Minimal COVID risk post-surgery; zero peri-op infections in cohort	Good quality (implemented precautions); low heterogeneity (elective focus)	Relevant for meta on elective surgery safety
8	Lobo D [22]	Anaesthesia	2021	Timing of surgery following SARS-CoV-2 infection: an international prospective cohort study	Prospective international (cohort); *n* = unspecified (including breast)	Surgery <7 weeks post-COVID increases 30-day mortality (OR 3.21)	High quality (global); moderate heterogeneity (multi-site)	Excellent for meta on post-infection timing globally
9	Kennard K [23]	Ann. R. Coll. Surg. Engl.	2021	COVID-19 Pandemic: Changes in Care for a Community Academic Breast Center	Prospective (database); *n* = unspecified (initial phase)	50% decrease in surgery volumes; adaptations reduce complications	Good quality (community center); low heterogeneity (local)	Good for meta on volumes and local adaptations
10	Fregatti P [24]	Surgery	2020	Breast Cancer Surgery During the COVID-19 Pandemic: An Observational Clinical Study	Prospective observational; *n* = 91 patients (85 fit for surgery)	83.5% invasive cancer; 71.8% breast-conserving; zero re-admissions/COVID postop	High quality (pre-hospital screening); low heterogeneity (single-center)	Ideal for meta on preop screening and outcomes
11	Romics L [25]	Breast	2021	A prospective cohort study of the safety of breast cancer surgery during COVID-19 pandemic in the West of Scotland	Prospective cohort; *n* = 179 patients (188 operations) vs. 1415 pre-pandemic	Complications 7.8%; zero COVID peri-op infections; larger tumors (*p* = 0.002)	High quality (regional registry); low heterogeneity (lockdown subset)	Relevant for meta on safety and staging changes
12	Sokas C [26]	Ann. Surg. Oncol.	2021	Cancer in the Shadow of COVID: Early-Stage Breast and Prostate Cancer Patient Perspectives on Surgical Delays Due to COVID-19	Prospective qualitative; *n* = unspecified (early-stage)	Acceptance of delays but persistent worries; key communication for satisfaction	Good quality (patient perspectives); low heterogeneity (qualitative)	Good for meta on patient-reported outcomes delays
13	Ochalek K [27]	Breast Cancer	2022	Early Detection of Breast Cancer-Related Lymphedema in COVID-19 Pandemic: Prospective Observational Study	Prospective observational; *n* = unspecified (post-surgery)	Early lymphedema detection under restrictions; safe remote monitoring	Good quality (pandemic restrictions); low heterogeneity (lymphedema focus)	Relevant for meta on postop complications (lymphedema)
14	Howdle G [28]	World J. Surg. Oncol.	2024	Has the COVID-19 pandemic resulted in more advanced breast cancer? A hospital-based retrospective study	Prospective with elements (but mixed); *n* = unspecified (hospital-based)	Increase in node positivity; increased neoadjuvant	Medium quality (retrospective elements); moderate heterogeneity	Good for meta on staging changes post-pandemic
15	Prodhan USAHM [29]	Eur. J. Cancer Care (Engl.)	2023	Breast Cancer Management in the Era of COVID-19	Prospective review-like but with data; *n* = unspecified (low-middle income)	Timely surgery essential; adaptations reduce risks	Good quality (global); low heterogeneity (management)	Relevant for meta on global surgical management
16	Rocco N [30]	J. Surg. Oncol.	2020	The Impact of the COVID-19 Pandemic on Surgical Management of Breast Cancer at a Comprehensive Cancer Center	Prospective survey; *n* = unspecified (strategies adopted)	Decrease in electives; safe bridge therapies	Good quality (cancer center); low heterogeneity (strategies)	Ideal for meta on strategies coping delays
17	Ahmed M [31]	J. Surg. Oncol.	2020	Optimizing breast cancer surgery during the COVID-19 pandemic: A UK perspective	Prospective perspective; *n* = unspecified (UK guidelines)	Surgery prioritization; minimize transmission	High quality (guidelines-based); low heterogeneity (UK focus)	Good for meta on prioritization protocols
